# Water Film-Driven Brucite Nanosheet Growth and Stacking

**DOI:** 10.1021/acs.langmuir.3c01411

**Published:** 2023-07-24

**Authors:** N. Tan Luong, Jean-François Boily

**Affiliations:** Department of Chemistry, Umeå University, Umeå SE 901 87, Sweden

## Abstract

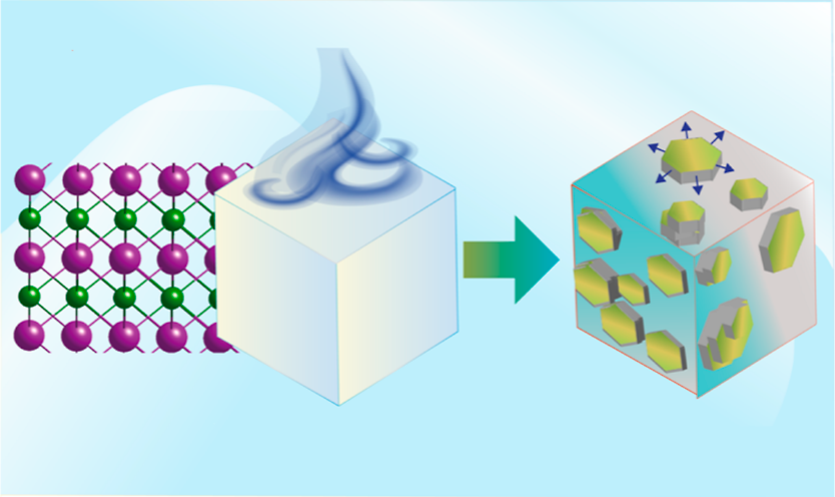

Thin water films that form by the adhesion and condensation of air moisture on minerals can initiate phase transformation reactions with broad implications in nature and technology. We here show important effects of water film coverages on reaction rates and products during the transformation of periclase (MgO) nanocubes to brucite [Mg(OH)_2_] nanosheets. Using vibrational spectroscopy, we found that the first minutes to hours of Mg(OH)_2_ growth followed first-order kinetics, with rates scaling with water loadings. Growth was tightly linked to periclase surface hydration and to the formation of a brucite precursor solid, akin to poorly stacked/dislocated nanosheets. These nanosheets were the predominant forms of Mg(OH)_2_ growth in the 2D-like hydration environments of sub-monolayer water films, which formed below ∼50% relative humidity (RH). From molecular simulations, we infer that reactions may have been facilitated near surface defects where sub-monolayer films preferentially accumulated. In contrast, the 3D-like hydration environment of multilayered water films promoted brucite nanoparticle formation by enhancing Mg(OH)_2_ nanosheet growth and stacking rates and yields. From the structural similarity of periclase and brucite to other metal (hydr)oxide minerals, this concept of contrasting nanosheet growth should even be applicable for explaining water film-driven mineralogical transformations on other related nanominerals.

## Introduction

Mineral nanoparticles capture air moisture in the form of water films that can be only a few monolayers (MLs) thick.^1−3^ These form nanoscale hydration environments that can alter the composition, structure, and functional properties of materials in unique, yet still misunderstood, ways.^2−7^ In particular, knowledge of (nano)coating growth within the confines of these films is essential to understand how exposure to air moisture alters mineral reactivity. This knowledge is of especial importance to atmospheric chemistry, catalysis, electrochemistry, environmental chemistry, geochemistry, and surface science.^8^

**Figure 1:**
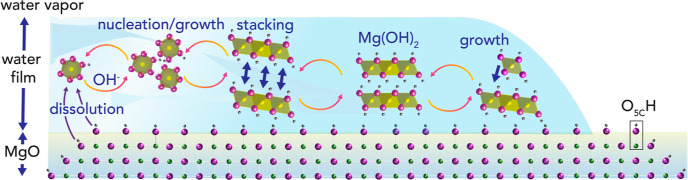
Brucite nanosheet growth from periclase in nanometric water films. Schematic representation of brucite growth involving Mg^2+^ dissolution, nucleation, nanosheet stacking, and growth to brucite. Water films are stabilized through interactions with surface OH sites (O_5C_H; O_5C_ is a pentacoordinated O) on ideal surfaces.

The conversion of the face-centered cubic structure of periclase (MgO; magnesia)^9,10^ to stacked Mg(OH)_2_ nanosheets in brucite [Figure 1; MgO + H_2_O → Mg(OH)_2_] is an ideal reaction that can help advance new ideas^6,11−18^ on mineral growth in molecularly thick water films. This reaction can also be of especial interest for ongoing and future applications of periclase-bearing materials and related metal oxides.^19−22^ Applications include steel and cement production,^20^ remediation engineering,^23−25^ refractories,^22^ flame retardants,^26^ antibacterial activity,^27^ as well as emerging technologies using metal oxide-bearing wastes for direct atmospheric CO_2_ capture.^28^

Transformations at pristine, Mg- and O- terminated periclase (nano)cube surfaces begin with the dissociative binding of water at Mg^2+^ and O^2–^ sites. Oxygen sites can be five-coordinated (5C) at pristine (100) surfaces (H_2_O + Mg_5C_^2+^–O_5C_^2–^ → Mg_5C_OH^+^–O_5C_H^–^)^29−32^ or of lower coordination at defects (e.g., O_4C_ at edges, O_3C_ at corners). These reactions produce OH-terminated surfaces in MgO (nano)particles, even those contacted to low levels of atmospheric humidity.^31^ This implies that most MgO surfaces outside vacuum conditions are predominantly OH-terminated or even, possibly, Mg(OH)_2_-like.^31^ When exposed to liquid water, these OH-bearing MgO (nano)particles readily convert to Mg(OH)_2_ via dissolution, Mg^2+^ hydrolysis, nucleation, and (crystal) growth.^33,34^ Such reactions can, also be driven in water films produced by the condensation of atmospheric moisture, provided that they are sufficiently thick to host liquid water-like hydration environments.

In recent work from our group,^35^ we resolved the transformation of MgO nanocubes to Mg(OH)_2_ nanoparticles in water films as thin as 3 MLs. These films were formed by exposing MgO nanocubes to a flow of N_2_(g) to 90% relative humidity (RH) at 25 °C. We showed that brucite growth was initiated by a solvent-driven nucleation-limited regime, in which growth was limited by competing ingestion and merging of Mg(OH)_2_ nucleation clusters (Figure 1). While this regime completely transformed 8 nm-wide MgO nanocubes to brucite nanosheets, reactions involving larger (32 nm-wide)^35^ nanocubes became diffusion-limited as Mg(OH)_2_ nanocoatings hampered the flux of reactive species to growth fronts.

Focus on the early, nucleation-limited, stages of Mg(OH)_2_ formation is of especial benefit for advancing ideas on mechanisms triggering crystal growth within the confines of water films (Figure 1). A crucial aspect to consider involves the conversion of Mg(OH)_2_ nanosheets to brucite, a process that requires stacking of individual nanosheets and/or step-wise (lateral and epitaxial-like) growth of hydrolyzed Mg^2+^ species at nanosheet surfaces. To this end, we hypothesized that water film thickness controls nucleation-limited growth. While 3D-like multilayered water films could favor growth by stacking, 2D-like sub-ML films could favor lateral or layer-by-layer growth as water films grow on newly-formed Mg(OH)_2_ surfaces.

In this study, we resolved water loading-dependent growth of Mg(OH)_2_ nanosheets by tracking the early stages of periclase–water film interactions. This was achieved by detecting the co-evolution of surface and bulk OH species using vibrational spectroscopy. Work with nanosized (8 nm-wide) MgO particles ensured the spectroscopic throughput needed to track low densities of OH sites needed to identify growth rates. Based on these measurements, we suggest that nanosheet precursors to brucite can only grow laterally in isolated patches of (2D-like) water films on periclase. Thicker water films, in contrast, facilitate nanosheet attachment and, therefore, the growth of brucite nanoparticles. These findings thus add insight into the impact of humidity on the early stages of layered mineral growth within the confines of molecularly-thick water films. This insight can, additionally, be applied for understanding water film-driven growth of other low-temperature (layered) materials of importance to nature and technology.

## Methods

### Periclase Synthesis and Characterization

Periclase (MgO) nanocubes were made by thermal dehydroxylation of synthetic brucite (Mg(OH)_2_, Figures S1–S3) at 500 °C for 2 h under ambient atmosphere. The resulting periclase powder was cooled down to 25 °C and then stored in a glove box (∼18 ppm H_2_O) to minimize exposure to atmospheric moisture and carbon dioxide. The brucite used to produce periclase was, in turn, synthesized by neutralizing a 0.2 M MgCl_2_ aqueous solution by a NaOH solution under a flow of N_2_(g). It was then repeatedly washed with MilliQ water to remove spectator ions and then dried at room temperature in N_2_(g). ^18^O-labeled brucite [Mg(^18^OH)_2_] was prepared by reacting 20 mg of periclase in 50 mL H_2_^18^O for 5 h. The product was then dried under a stream of N_2_(g). All dry materials were then ground to a powder using a mortar and pestle.

Physicochemical properties of the non-isotopically exchanged [Mg(^16^OH)_2_] periclase samples are reported in Table S1. Phase purity was confirmed by powder X-ray diffraction (XRD) in the 10–90° 2θ range using a PANalytical X’Pert^3^ powder diffractometer (1.54187 Å Cu Kα radiation at 45 kV and 40 mA) operating under reflection mode. Particle size and morphology were assessed by scanning electron microscopy (SEM) and bright-field transmission electron microscopy (TEM) imaging. SEM images were taken on a Carl Zeiss Merlin microscope, while a FEI Talos L120 microscope (120 kV) was used for low-resolution TEM images. Brunauer–Emmet–Teller (BET) specific surface area, Barrett–Joyner–Halenda (BJH) pore size, and volume were obtained from 90-point N_2_(g) adsorption/desorption isotherms. These isotherms were collected on samples previously degassed at 110 °C under a flow of N_2_(g) for 24 h using a Micromeritics TriStar 3000 instrument. Finally, the elemental composition of the periclase nanocube surfaces was identified by X-ray photoelectron spectroscopy (XPS, Kratos Axis Ultra electron spectrometer equipped with Al Kα X-ray source, 150 W, and a delay line detector). Here, survey spectra were collected from 0 to 1100 eV at a pass energy of 160 eV, while core level spectra of C 1s, O 1s, and Mg 2p were taken at 20 eV.

### Initial Water Loadings on Periclase

Water loadings achieved in the initial stages of exposing water vapor to periclase were determined by microgravimetry, using a DVS Advantage ET 2 instrument (Surface Measurement Systems). Measurements were acquired through a 11-point adsorption isotherm on a 21.656 mg periclase sample exposed to 0 and 95% RH at 25 °C. This sample was initially dried with N_2_(g) (0 RH) for 5 h. The equilibrium criterion for each preselected RH level was 60 min and a complete adsorption isotherm took up to 10 h.

### Humidity-Driven Transformations

Periclase hydroxylation reactions induced by thin water films were monitored by Fourier transform infrared (FTIR) spectroscopy. These reactions were induced by exposing periclase to a flow of 500 mL/min N_2_(g) at prelected values of humidity in 10–90% RH range and for periods of up to 58 h. This gas composition was prepared using a humidity generator module (proUmid MHG32). Fresh periclase samples were used for every chosen experiment. In an additional set of experiments, isotopic labeling reactions were performed by exposing samples to a stream of 200 mL min^–1^ N_2_ (g) carrying 10–70% RH ^2^H_2_O or H_2_^18^O vapor. This gas composition was prepared by mixing a stream of N_2_(g) saturated with ^2^H- or ^18^O-labeled water with another dry stream of N_2_(g) using mass flow controllers (MKS, 179A).

FTIR spectra were collected in transmission mode on (∼2–5 mg) periclase samples pressed onto a fine tungsten mesh (Unique Wire Weaving, 0.002 in. mesh diameter). This mesh was, in turn, held with a copper sample holder in direct contact with a K-type thermocouple. The sample holder was placed in the middle of an optical reaction chamber (AABSPEC #2000-A) equipped with CaF_2_ windows. The samples were first outgassed for 2 h in vacuo (<2.5 mTorr, the detection limit of capacitance manometer; MKS, Baratron) and then exposed to moist N_2_(g). All spectra were acquired at a resolution of 4 cm^–1^ over the 600–4500 cm^–1^ range at forward/reverse scanning rate of 10 kHz and obtained by co-adding 100 spectra every 89 s. All measurements were carried out using a Bruker Vertex 70/V instrument equipped with a deuterated l-alanine doped triglycine sulfate (DLaTGS) detector.

The wavenumber (ν)-dependent absorbances (*A*) of the O–H stretching and water-bending regions were modeled using a linear combination of Gaussian components
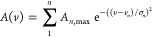
1Here, *A*_*n*,max_ is the maximal absorbance of the *n*th component centered at wavenumber ν_*n*_ and with width of the distribution σ_*n*_. This method was preferred for this work because attempts using multivariate methods^36^ could not clearly tease out the response of the evolution of different bands over reaction time. Spectral deconvolution was performed in a sequence of time-resolved spectra with absorbance values offset to 0 in the 3800–4000 cm^–1^ range for the analysis of a portion of the O–H stretching region (3650–3800 cm^–1^), and in the 1700–1800 cm^–1^ range, for the analysis of the water bending region. To account for differences in sample mass in different experiments, all absorbance values were normalized to that of the unreacted periclase at 3600 cm^–1^. Time-resolved Gaussian component absorbance growth were then modeled using a 1st-order kinetic equation [*A*(*t*) = *A*(*t* = ∞) (1 – e^–*kt*^); *A*(*t* = ∞) is absorbance at the end of the reaction at time *t* = ∞, and *k* is the constant]. Absorbance losses were modeled using a first order decay model [*A*(*t*) = *I*(*t* = 0) e^–*kt*^; *A*(*t* = 0) is initial band absorbance, and *k* is the constant]. All Gaussian and kinetic parameter optimization (non-linear least square) calculations were performed with MATLAB (version R2021b, The Mathworks, Inc.).

### Molecular Dynamics

Molecular dynamics (MD) simulations of two defect (100) faces of periclase were performed using GROMACS/2021.1.^37^ Simulation cells consisted of a 3.03 × 3.03 × 3.03 nm periclase nanocubes with two opposite surfaces containing (i) two monoatomically deep troughs terminated by OH groups at doubly coordinated (O_2C_) and quadruply coordinated (O_4C_) sites, and (ii) one monoatomically thick MgO cluster containing 8 Mg^2+^ sites surrounded by hydroxyls at O_2C_ and O_4C_ sites. The (100) surfaces were exposed to a 10 nm-thick vacuum in which 10 to 400 water molecules were randomly inserted.

Simulations were carried out using the Clayff^38^ force field. A NVT [constant number (*N*) of particles, constant volume (*V*), and constant temperature (*T*)] ensemble and a time step of 1.0 fs were used with the Verlet algorithm^39^ to integrate the equations of motions for all the atoms in the system, which were projected using a periodic boundary condition. The temperature of the system (300 K) was coupled to the Nosé–Hoover^40^ velocity-rescale thermostat with a 0.1 ps relaxation time. The O–H bond strength of all the hydroxyls were treated by the LINCS^41^ algorithm. A 0.8 nm cutoff was used for non-bonded van der Waals interactions, and the particle-mesh Ewald^42^ method was used to treat long-range electrostatic interactions.

Simulation cells were first energy-minimized (double precision) using a steepest descent algorithm. The resulting structure was then equilibrated (single precision) using classical MD for at least 10^7^ steps (10 ns), followed by production runs of at least another 10 ns. Total energy convergence and its components, as well as temperature and atomic densities, were monitored for these entire equilibration periods. Water-periclase O(H) hydrogen bond analyses, Mg–O contact pairs, and water density maps were calculated using utilities of GROMACS/2021.1.^37^

## Results and Discussions

**Figure 2:**
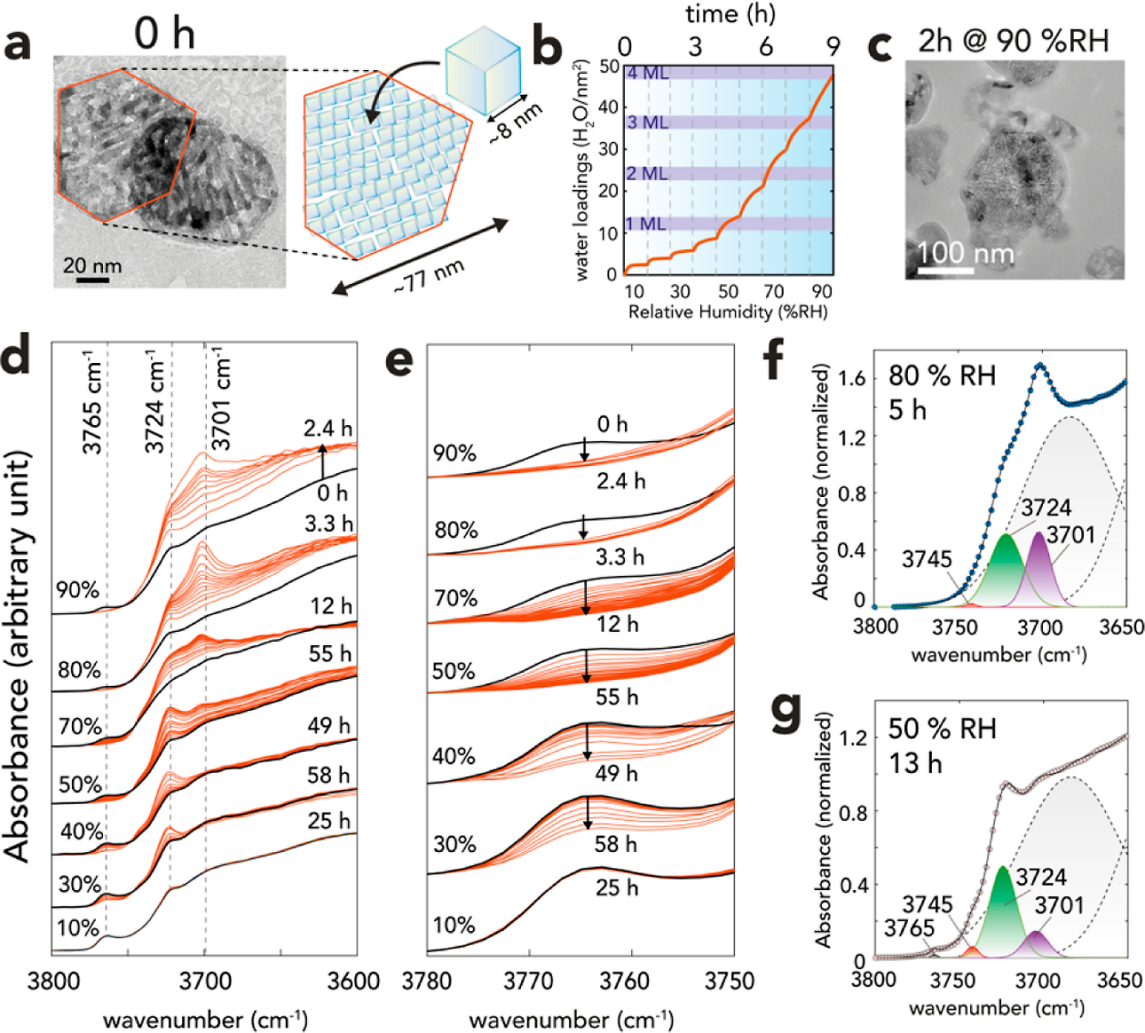
Humidity-driven transformations of periclase to brucite. (a) Transmission electron microscope imaging of ∼8 nm-wide periclase nanocubes clustered in hexagonal casings, which are relicts of synthetic brucite from which they were produced. (b) Microgravimetrically derived water loadings acquired by exposing periclase from 10 to 90% RH, with 1 h of reaction time at each pre-selected humidity level. Equivalent water loadings in ML (1 ML = 12 H_2_O/nm^2^) are the result of water film formation and Mg^2+^ hydrolysis. (c) Imaging of periclase reacted at 90% RH for 2 h. (d,e) Time-resolved vibrational spectra of periclase nanocubes exposed to a flow of N_2_(g) with 10–90% RH at 25 °C. The O–H stretching region revealing OH groups in periclase (black lines) and in the reacted materials (orange lines), notably brucite at 3701 cm^–1^. Reported spectra were selections of those collected at an interval of 89 s. (f,g) Examples of spectral deconvolution (data = circle symbols; model = lines) using a linear combination of Gaussian-shaped spectral components for surface (3745, 3765 cm^–1^) and bulk (3401, 3724 cm^–1^) OH groups, alongside other bulk background contributions at lower O–H stretching frequencies (dashed lines) from non-stoichiometric bulk OH groups of periclase.

Early stages of brucite growth were studied by exposing synthetic periclase nanocubes to a stream of water vapor (Figure 2). The (∼8 nm-wide) nanocubes chosen for this work were aggregated in 77 ± 25 nm-wide two-dimensional hexagonal casings (Figure 2a; cf. Figures S1–S5 and Table S1 for characterization),^43^ which were relicts for brucite nanoparticles from which they were synthesized. This aggregation pattern emerged from thermal hydroxylation reactions which, as we explained in our previous work,^35^ produced a 2D array of periclase nanocubes. As such, the considerable volumetric compression (∼67%; ρ_periclase_ = 3.5 g cm^–3^; ρ_brucite_ = 2.3–2.4 g cm^–3^) undergone by the materials left a maze-like microporous network (Figure S5). For reference, in an idealized 2D array of particles, this would amount to an empty space of ∼4 nm around each single nanocube surfaces. In this study, we resolved the early stages of hydroxylation reactions back to brucite by reacting these ∼8 nm wide nanocubes with water films of up to ∼3 ML (Figure 2b).

From microgravimetry (Figure 2b), we show that sub-ML films formed under up to ∼50% RH. These loadings rapidly reached near-equilibrium as water vapor adsorbed on periclase, then condensed as water films. These loadings were, additionally, stable for at least 1 h of reaction time. In contrast, exposure to films of at least ∼1 ML, produced by exposing the particles to higher levels of humidity (>50% RH), triggered a continual uptake of water over reaction time. While the initial stages of uptake were also the result of water vapor condensation on the particles, the prolonged uptake was driven by Mg^2+^ hydrolysis reactions within the confines of the films.^35^ This was confirmed by vibrational spectroscopy (Figure 2d–g) through the continual growth new O–H stretching bands signaling the formation of new OH-bearing species. Still, these early stages of the reactions altered only the topmost portion of the nanocubes because (i) we previously^35^ showed that reactions at 90% RH for 2 h produced only minute quantities of crystalline brucite and (ii) particle morphologies remained unchanged (Figure 2c; cf. Figure 5a of Luong et al.^35^ for comparison).

**Figure 3:**
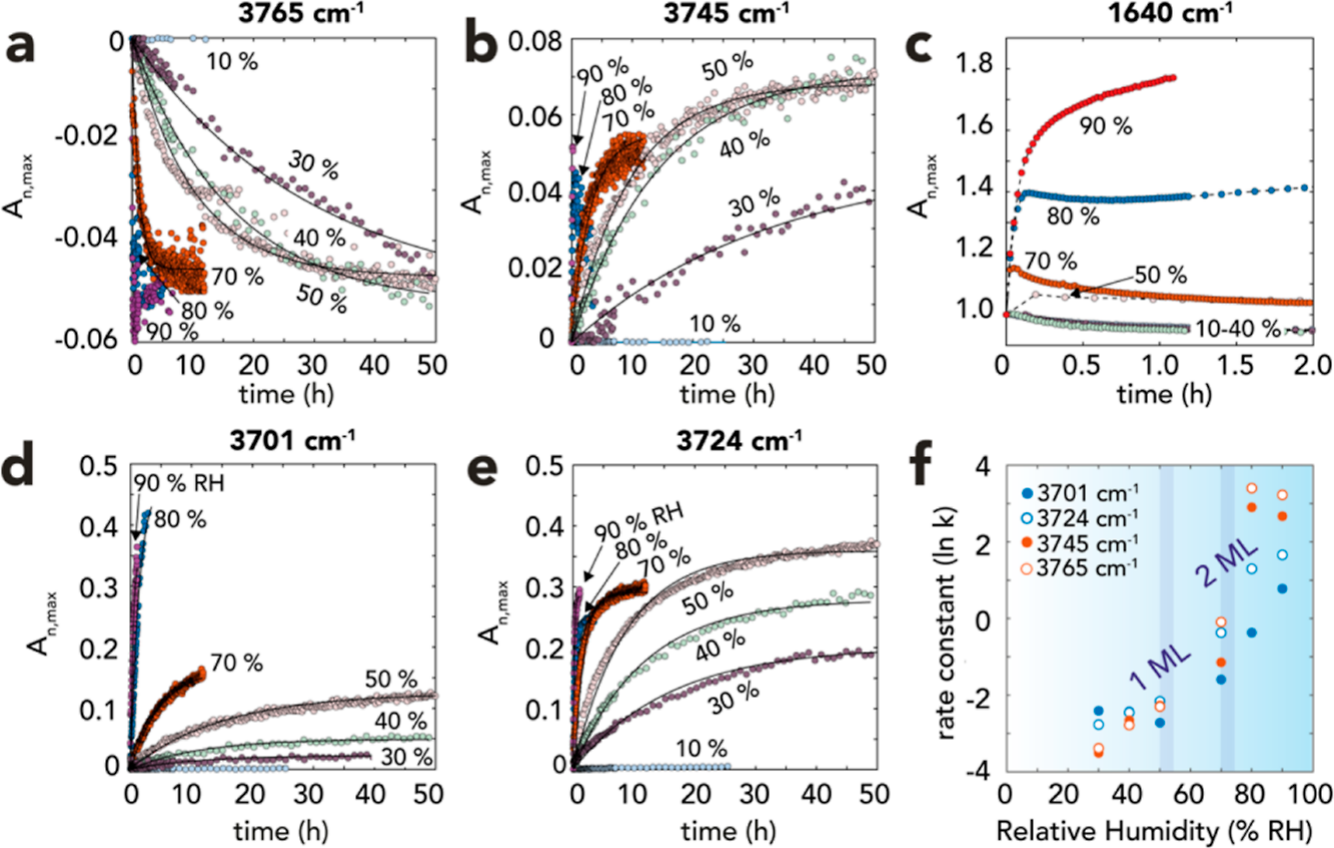
Gaussian deconvolution analysis and kinetic modeling of data in Figure 2d,e uncovering the evolution of OH species and water over time and humidity. (a–e) Component absorbances (*A*_*n*,max_; eq 1) of (a,b) surface OH groups, (c) water film (Figure S6), (d,e) bulk OH groups. Absorbance losses of (a) the 3765 cm^–1^ band were recovered through (b) the 3745 cm^–1^ band, as a result hydrogen bond formation with first layer water molecules. (c) The water bending band (Figure S6) revealed an initial loss of water at low (≤70% RH) and steady or increasing loadings at high (≥80% RH) humidity. (d,e). All lines in (a,b,d,e) were first-order kinetic model predictions. (f) First-order rate constants (ln *k*) of data for OH groups (a,b,d,e) with respect to % RH.

To identify the time- and humidity-dependence of these new OH species, we resolved the O–H stretching (Figure 2d,f–g) and water bending region (Figures 3c and S6) using Gaussian deconvolution (eq 1; Figure 3). This procedure extracted information on the hydration of periclase surface (Figure 3a–c), and the concomitant evolution of hydroxylated Mg^2+^ species (Figure 3d,e), including the flagship band of brucite at 3701 cm^–1^ (Figure 3e). Kinetic modeling of time-resolved Gaussian absorbances revealed congruent humidity-dependent (1st-order kinetic) rates (ln *k*) for all OH species, with values scaling with humidity. This relationship underscored a tight coupling between water film coverages and brucite formation rates and yields. We highlight this link in the following two sections, first by describing water film growth on periclase nanocubes, then by explaining the water-dependent growth of brucite nanosheets.

### Periclase Surface Hydration

To explain the early stages of brucite growth, we began by resolving spectral evidence for the establishment of water films on periclase nanocubes. This was provided by the response of the well-known O–H stretching band^29−32^ (3765 cm^–1^) of periclase surface OH groups (Figures 2e and 3a). These are groups that were already present on periclase prior the reactions, as also evidenced by XPS (Figure S4). Based on the frequency of this band, we expect that these groups were singly- (O_1C_) and/or doubly- (O_2C_) coordinated with underlying Mg^2+^ sites.^29−32^ Additionally, from the Gaussian-like shape and narrow width (i.e., low half-width-at-half-maximum) of the band, these OH groups are expected to be located at highly specific coordination environments at particle surfaces.^29^ Considering high resolution images^35^ (Figure S2d) confirming the crystallinity of the MgO nanocubes, we infer that these specific bonding environments were at crystallographic sites.

Gaussian deconvolution of the O–H stretching region (Figure 3) revealed that water films shifted the vibrational frequency of surface OH groups (Figure 3a) to a new band at 3745 cm^–1^ (Figure 3b). This shift can be explained from the highly sensitive response (125–175 cm^–1^/pm)^44−47^ of the O–H bond strength of these groups to hydrogen bonds established with first layer film water molecules. The relationship between these two bands can also be appreciated by (i) the recovery of absorbance losses at 3765 cm^–1^ by the new band at 3745 cm^–1^ (Figure 3b) and (ii) their congruent humidity-dependent (1st-order kinetic) loss (3765 cm^–1^) and growth (3745 cm^–1^) rates (Figure 3f). Additionally, the narrow width of the resulting 3745 cm^–1^ band (Figure 3e) implies that highly specific hydrogen bonded environments were also formed with these OH groups.

Thus, taking the 3765 cm^–1^ band (Figures 2b and 3a,b) as a primary marker for hydration, we find that particle surfaces were completely hydrated within a few minutes of reaction at ≥80% RH, but only after ∼5 h at 70% and ∼50 h at 30–50% RH. To explain this result, we first considered the possibility that a portion of the sites could have been at buried interfaces and/or within (nano/micro)pores, as water could take more time to diffuse into these environments. We, however, discard these possibilities because N_2_(g) adsorption/desorption (Figure S5 and Table S1) provides no evidence for considerable levels of intraparticle microporosity. Additionally, such buried or pore OH sites are not likely to have generated the narrow 3745 cm^–1^ band but rather broad bands reflecting a variety of coordination (hydrogen- and metal-bonding) environments, which are expected at disorganized mineral surfaces.

An alternative explanation is the possibility for an inhomogeneous distribution of water films on periclase nanocubes. This possibility is supported by recent imaging efforts from our group,^5^ suggesting thatwater films can preferentially accumulate at particles edges and along defects. This phenomenon is, however, preferentially manifested only at low % RH, where water films form via direct (adhesive) binding to mineral surface sites. From the time-resolved absorbances of the bending mode of water (1640 cm^–1^) in Figure 3c, we find that water film loadings in the first minutes to hours of reaction at ≤70% RH were affected by competing adsorption/condensation and hydrolysis reactions. We arrive at this interpretation by noting that, following an initial hike in loadings from water vapor binding, loadings progressively decreased over 1–2 h (Figure 3c). This decline in film coverage can be taken as evidence that rates of Mg^2+^-driven hydrolysis of water OH-bearing species exceeded those of water film growth. This mechanism may have consequently drawn water films to active regions of hydrolysis, and it supports the idea for coexisting hydrated and dehydrated regions on periclase.

**Figure 4:**
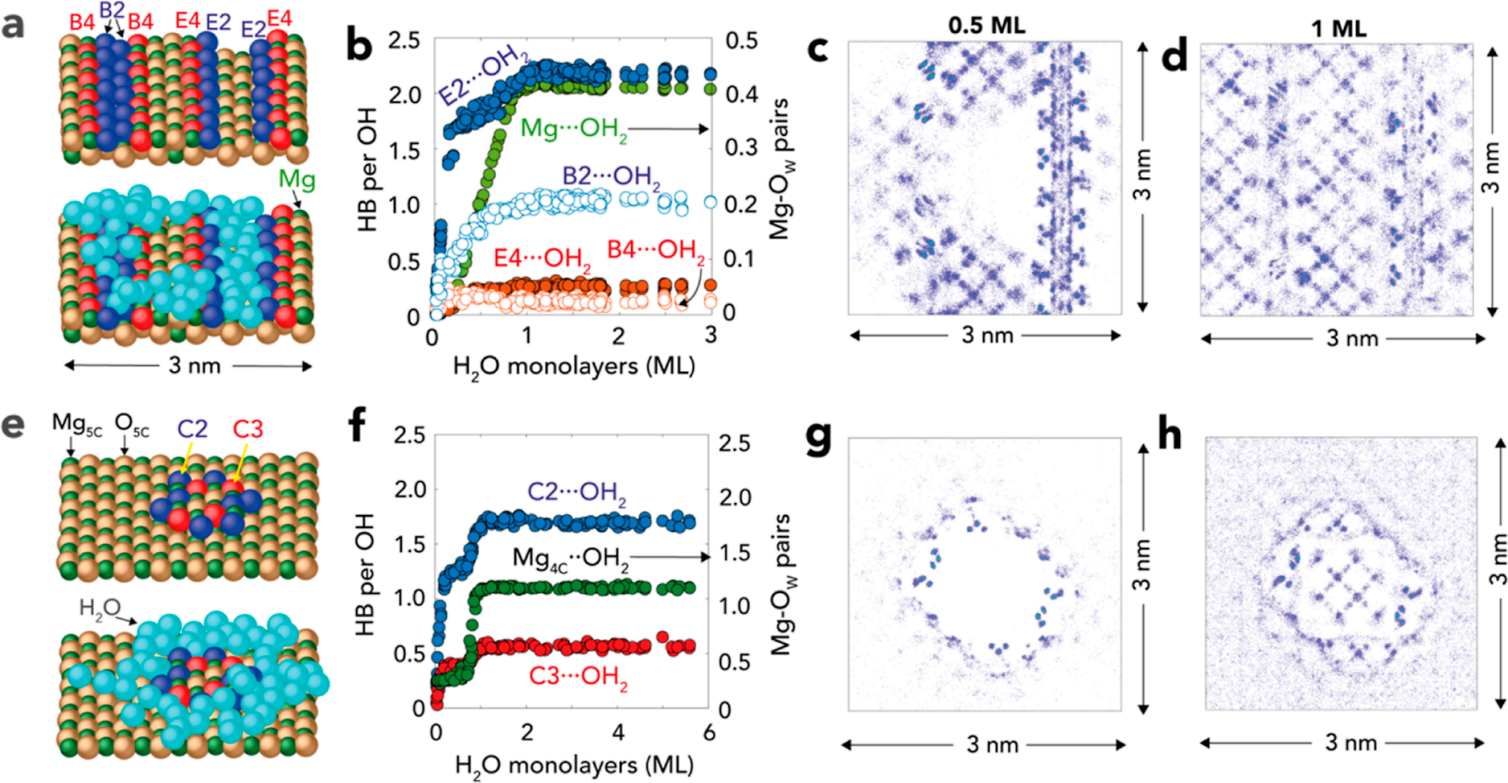
Molecular simulations revealed inhomogeneous distributions of sub-ML water films. MD simulations (10^7^ steps at 1.0 fs time steps using CLAYFF^38^) of the hydration of a defective (100) face. (a,e) Surface of 3 × 3 × 3 nm simulation cells showing (top) dry and (bottom) hydrated surface, exposing (a) rows of doubly coordinated (O_2C_; B2, E2) and quadruply coordinated (O_4C_; E4, B4) hydroxyl groups along the edges of monoatomically deep troughs and (e) a monoatomically thick hydroxylated MgO nanocluster exposing oxygen corners (C2, C3) and quadruply coordinated Mg^2+^ (Mg_4C_) on a flat (100) face exposing pentacoordinated Mg^2+^ (Mg_5C_) and O^2–^ (O_5C_) sites. (Mg = green, O = yellow, OH = red and blue; H_2_O = turquoise). (b,f) Hydrogen bond (HB) number of sites (left ordinate axis) and Mg–OH_2_ contact pairs (right ordinate axis) as a function of H_2_O MLs. (c,d,g,h) Surface density maps of the surfaces shown in (a,e) at (c,g) sub-ML and (d,h) 1 ML coverage.

Because this concept of inhomogeneous films coverage could only be inferred by coexisting spectroscopic markers for dehydrated (3765 cm^–1^) and hydrated (3745 cm^–1^) bands, we used MD to offer a visual depiction of plausible scenarios. To this end, we simulated MgO surfaces exposing corners and edges, which are well-known^48,49^ sources of OH sites of low Mg-coordination number generating the narrow 3765 cm^–1^ band. We simulated open, defect-free, (100) MgO surfaces alongside (i) open (E2) and buried (B2) monoatomically deep edges exposing OH groups of low coordination (Figure 4a–d) and (ii) a monoatomically thick hydroxylated MgO nanocluster exposing corners (C2, C3) and quadruply coordinated Mg^2+^ (Mg_4C_) sites (Figure 4e–h). Both surfaces exposed penta-coordinated Mg_5C_ and O_5C_ sites on neighboring defect-free regions. While a variety of other defects were certainly possible, consideration of these two model surfaces was sufficient to depict the general idea of how even the mildest defects impact spatial distributions of water.

These simulations confirmed that (i) water preferentially attached to defect OH sites, and that (ii) cohesive water–water interactions favored water condensation to these regions (Figure 4c,g). This preferential accumulation was, however, only detected in sub-ML thickness because defects mostly affected the distribution of first layer water molecules. This implies that all surface OH groups were completely hydrated in films exceeding 1 ML, namely where maximal number of Mg_5C_···OH_2_ in the defect-free regions were reached. These findings consequently fall in line with our experimental results that revealed coexisting unhydrated and hydrated OH groups in conditions where water films were less than ∼1 ML thick, namely in up to ∼50% RH (Figure 2b).

From these experimental and modeling results, we suggest that the early stages of MgO-water interactions produced inhomogeneous distributions of water films in which Mg^2+^ hydrolysis reactions took place. The films, however, progressively migrated over the entire nanocube surfaces over reaction time as Mg(OH)_2_ nanosheets formed at the periclase surface. Building upon these findings, we can now describe the growth of brucite in the early stages of contact between periclase and water films.

### Brucite Growth by Nanosheet Stacking

The time-resolved Gaussian absorbances of the ∼3701 cm^–1^ band were described using a first-order kinetic model (Figure 3d). While brucite growth rates were relatively constant in films of less than ∼1 ML (Figure 3f), they became proportionally greater in thicker water films, increasing by ∼4 ln *k* units in films of up to ∼2 ML. This indicated that 3D-like hydration environments facilitated brucite growth. We also find that brucite growth rates were on par with those of the humidity-dependent surface hydration, here seen through establishment of hydrogen bonds with surface OH groups seen through the 3745 and 3765 cm^–1^ bands (Figure 3a,b). These comparable rates further confirmed a tight link between the progression of water film coverage and brucite growth.

**Figure 5:**
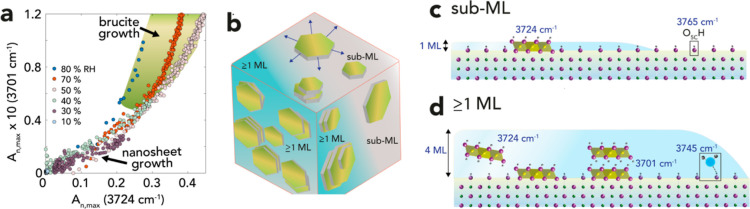
(a) A link between the nanosheet and brucite OH sites. Relationship between Gaussian component absorbances (*A*_*n*,max_) of the precursor (3724 cm^–1^) and brucite (3701 cm^–1^) components generated by reactions in 10–80% RH, except at 10% RH where reactions did not occur. The shaded area marks a range of conditions of accelerated brucite growth in multilayered water films. (b–d) Schematic representation of brucite nanosheet growth in sub-ML and multilayered water films. (b) A single periclase nanocube and within a (c) sub- to 1-ML and (d) multilayered water films. Sub-ML films sustain nanosheet lateral growth [cf. arrows in (b)] in isolated water films. Multilayered films (d) facilitated nanosheet stacking due to enhanced nanosheet mobility. Band assignments are for surface OH (3765 cm^–1^), hydrated surface OH (3745 cm^–1^), poorly stacked/unstacked nanosheets (3724 cm^–1^), and brucite (3701 cm^–1^). Atomic distances and water film thicknesses in (c,d) are to scale, except for the physisorbed water molecule (turquoise, 3745 cm^–1^).

Gaussian deconvolution of the spectra revealed the evolution of a fourth, and final, OH-bearing species through a sharp band at 3724 cm^–1^ (Figure 3e). Unlike brucite OH species (3701 cm^–1^), this species formed both sub-ML and thicker water films. It also formed at highly comparable humidity-dependent rates as brucite (Figure 3f). This can also be appreciated in Figure 5a, through the strong relationship between the absorbances of this species (3724 cm^–1^) and that of brucite (3701 cm^–1^). Although previously assigned to periclase surface OH groups,^50^ we conducted isotopic exchange experiments (Figures S7–S9) that instead suggest that the 3724 cm^–1^ band was from an OH-bearing solid. This was first confirmed by the resilience of this band to deuteration by D_2_O(g) (Figures S7–S9), a response that strongly contrasts with the immediate exchange undergone by surface OH groups (3765 cm^–1^).^51,52^ We support this interpretation by noting that surface groups are unlikely to be resilient to deuterium exchange, regardless of differences in coordination environment with respect to underlying Mg^2+^ sites or hydrogen bonding environment. We base this assertion from our previous deuterium exchange work^47,52^ on a variety of mineral surface OH groups. Second, we find that producing this OH species by exposing periclase to H_2_^18^O (g) (Figure S8c) induced an isotopic shift (Δν_O–H_ = 14 cm^–1^; 3724 → 3710 cm^–1^) in its stretching frequency that was highly comparable to the one observed in synthetic Mg(^18^OH)_2_ (Δν_O–H_ = 11 cm^–1^; 3701 → 3690 cm^–1^). Based on these findings, we concluded that these OH species belonged to a structurally related brucite precursor. However, contrarily to brucite, this precursor grew in both sub-ML and multilayered water films.

Building upon these results, we can propose the following mechanism for Mg(OH)_2_ nanosheet generation in the early stages of contact between periclase nanocubes and water films (Figure 5). We propose that the 3724 cm^–1^ band is from OH-bearing precursor solids consisting of poorly stacked or dislocated brucite nanosheets. These might even correspond to previously imaged^53,54^ growth products on periclase exposed to water vapor. When the humidity was well below 70% RH, these solids were more likely to grow laterally in isolated (2D-like) water films (Figure 5b,c). Additionally, the absence of brucite under these conditions indicated that water films were still insufficiently thick to sustain epitaxial-like growth through newly formed films on brucite nanosheets, at least in the early stages of the reactions.

In contrast, thicker (3D-like) water films formed at higher humidity greatly facilitated brucite growth (Figure 5d). We take this as indication that these thicker hydration environments could have driven growth by enhancing nanosheet mobility. This established direct intersheet hydrogen bonds that can weaken O–H bond strength and, therefore, explains the red-shift of the 3724 cm^–1^ band of the precursor solids to the 3701 cm^–1^ band of brucite. This can also be appreciated by the direct relationship between the absorbances of both 3724 and 3701 cm^–1^ bands (Figure 5a), up to a tipping point triggered by the thickest water films considered in this work (shaded area in Figure 5a), where brucite growth became the dominant Mg(OH)_2_ formation process. Additionally, the sustained growth of the 3724 cm^–1^ band in multilayered water films signals that hydrolysis continuously supplied poorly stacked/dislocated nanosheets needed for brucite growth. Here, the 3D hydration environments of these thicker films could have also secured fluxes of reactive species that sustained (e.g., lateral and epitaxial) growth in both nanosheet precursors and newly grown brucite particles on periclase.

## Conclusions

By capturing crucial moments in the initial stages of periclase–water interaction, this work provides insight into nanolayered brucite growth in molecularly thick water films. Through time-resolved spectroscopic information, we find that the first minutes to hours of brucite growth followed first-order kinetics, with rates scaling with water loadings. Growth was, additionally, tightly linked to periclase surface hydration, and to the formation of a brucite precursor solid akin to poorly stacked/dislocated nanosheets. This adds to a mounting body of evidence^17,55,56^ for amorphous/low-crystalline seed material of relatively high solubility leading to crystal growth of lower solubility.

Conditions of low humidity (<50% RH) formed sub-ML water films that could not sustain brucite growth, yet these produced precursor nanosheets. From our concept of film migration toward dehydrated regions over reaction time, it is possible that poorly stacked or dislocated nanosheets grew laterally in 2D-like patches of isolated water films. Although this could not be verified by imaging, and given the small reaction yields in these early stages of the reaction, it aligns with previous evidence for inhomogeneous distributions of water films on hydrophilic minerals.^5^ Conditions of high humidity (≥50% RH) facilitated, in contrast, the co-evolution of both precursor and brucite nanosheets in multilayered 3D water films. These conditions enhanced nanosheet mobility and promoted intersheet hydrogen bond formation without, however, producing sufficiently crystalline brucite in these early stage periclase–water interactions.

Our vibrational spectroscopic approach to track early stages of film-driven nanosheet growth enabled us to infer on mechanisms that cannot otherwise be directly captured by imaging . It could also have far-reaching implications for understanding transformations in nanosheet growth on other structurally- and chemically-related oxides of central importance to nature and technology.

## Supporting Information Available

Electron microscopy images, X-ray powder diffraction diffractograms, micropore analysis, X-ray photoelectron spectra of synthetic materials, and additional FTIR spectroscopy results of the water bending region and of isotopic exchange experiments (PDF)
