# Supplementary material for: Water Film-Driven Brucite Nanosheet Growth and Stacking
Source: Langmuir. 2023 Jul 24;39(31):11090–8. doi: 10.1021/acs.langmuir.3c01411 (PMC10413962; doi:10.1021/acs.langmuir.3c01411)
Supplement: Supplementary file 1 — la3c01411_si_001.pdf [file la3c01411_si_001.pdf]

SUPPLEMENTARY SECTION

Water film-driven brucite nanosheet growth and stacking

*N. Tan Luong and Jean-François Boily**

Department of Chemistry, Umeå University, SE 901 87 Umeå, Sweden

*****corresponding author: *jean-francois.boily@umu.se*

Table of Contents

Figures ____ 2
Fig. S1. ____ 2
Fig. S2. ____ 2
Fig. S3. ____ 2
Fig. S4. ____ 3
Fig. S5 ____ 3
Fig. S6. ____ 4
Fig S8 ____ 5
Fig S9 ____ 6
Table S1 ____ 7

Figures

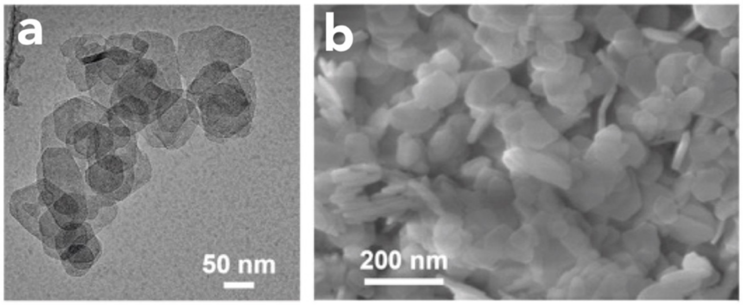

Fig. S1. Electron Microscopy imaging of synthetic brucite. TEM (a) and SEM (b) of original brucite before dehydroxylation.

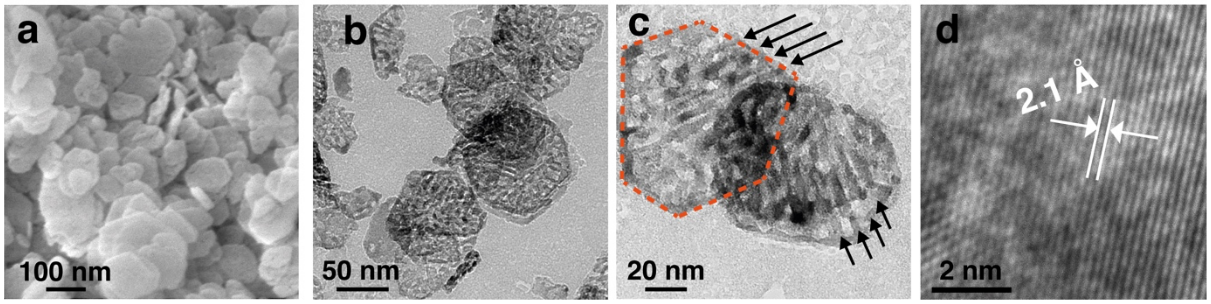

Fig. S2. Electron Microscopy imaging of periclase. (a) SEM and (b-d) TEM images of synthetic periclase particles, produced by the dehydroxylation of brucite (Fig. S1) at 500 °C for 2 h under ambient air.

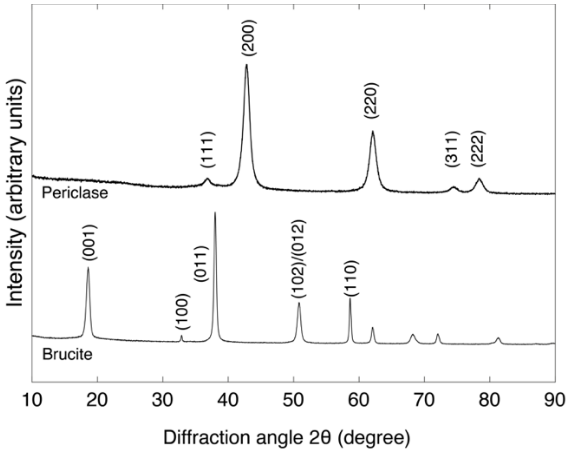

Fig. S3. Structural characterization. XRD profiles of periclase and brucite.

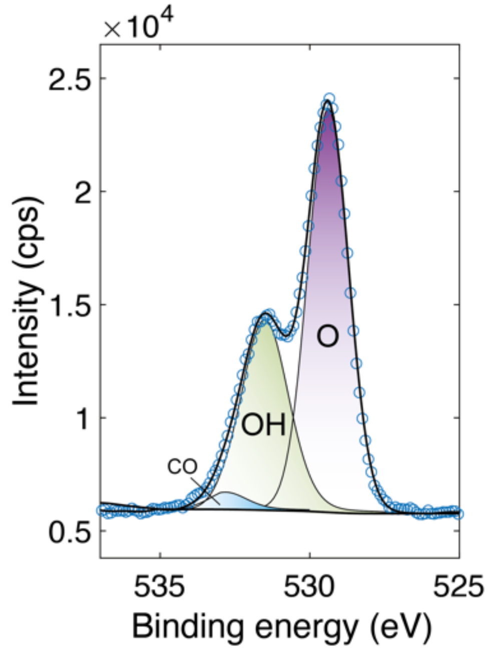

Fig. S4. Surface O composition of periclase by XPS. XPS O 1s spectra of fresh synthetic periclase. XPS retrieved an overall composition of $MgO{\cdot}(OH)_{0.5}$, and therefore the presence of 0.5 non-stoichiometric OH groups for every O. From the analysis depth (<10 nm) of the technique, this should reflect the composition of the entire ~8 nm wide nanocubes. These groups were also detected by vibrational spectroscopy through strongly (3745 cm^{-1}) hydrogen-bonded of the MgO bulk and isolate (3765 cm^{-1}) OH groups at the surface.

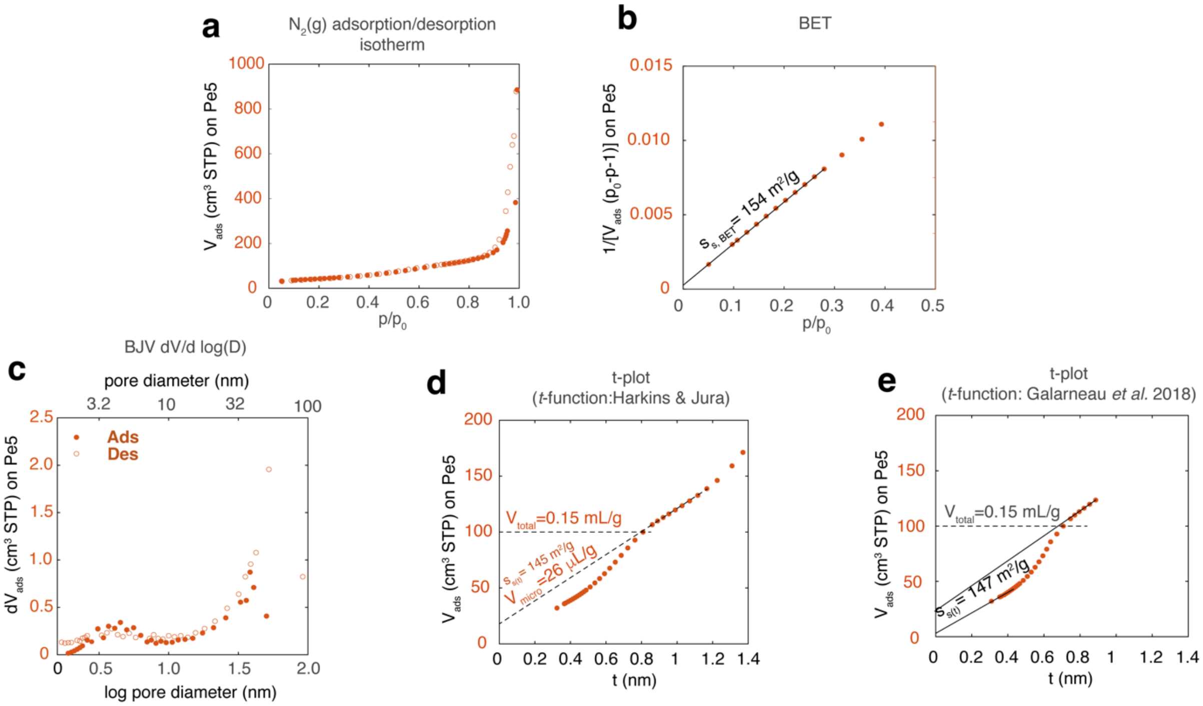

Fig. S5. N_2(g) adsorption/desorption isotherm results showing (a) raw data, (b) calculation of specific surface area by BET method, (c) BJH analyses and (d-e) t-plots analyses. These revealed specific surface area on par with particle sizes, and microporosity of 26 μL/g in Pe5. We assign this microporosity to interparticle voids, given their comparable distributions of values in nanocube assemblages.

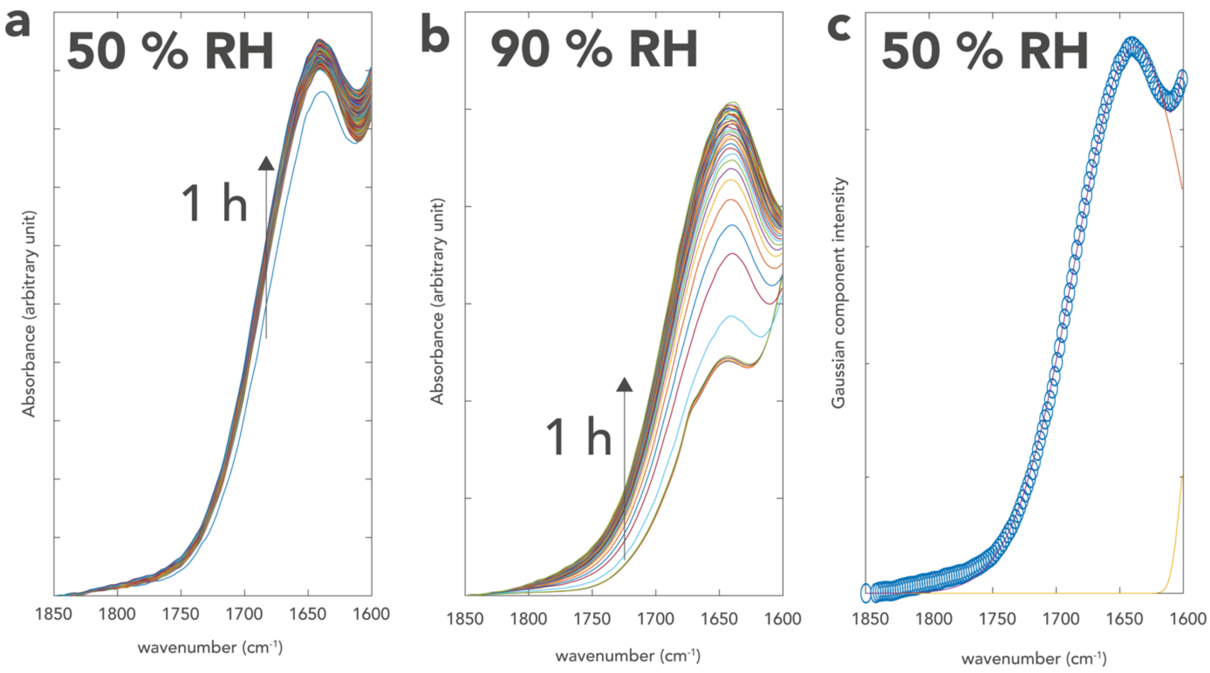

Fig. S6. Representative spectra of the water bending region. (a) 50 % RH and (b) 90 %RH over the first hour of reaction. (c) Gaussian deconvolution (red line) of the absorbances of a selected spectrum (blue open circles). Time-resolved Gaussian component intensities of the ~1640 cm-1 were used to resolve relative water coverages (cf. Fig. 3 of main text). All spectra were normalized for absorbance of the dry unreacted MgO at 3600 cm^{-1}.

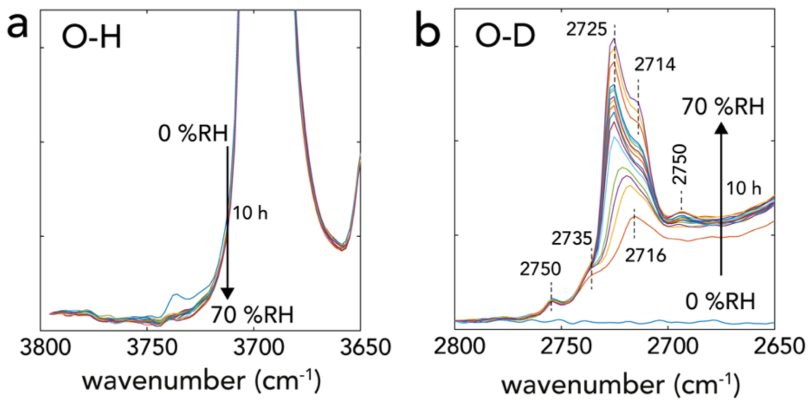

Fig. S7. Isotopic exchange revealing surface OH groups on synthetic brucite shows that these groups cannot explain the 3724 cm^{-1} band. FTIR spectra of the (a) O-H and (b) O-D stretching regions as synthetic brucite is exposed to 70% RH D_2O vapor over the course of 10 h. Surface OH groups on brucite (*e.g.*, non-stoichiometric OH, basal OH, edges OH and corner OH groups) are readily exchanged by exposure to D_2O vapor. These measurements revealed that there are no surface OH groups from brucite above 3740 cm^{-1}. The highest vibrational frequency from (possibly singly-coordinated (*i.e.,* –OH or O_{1C})) surface OH groups was detected at 3736 cm^{-1}, which was shifted to 2750 cm^{-1} by deuteriation.

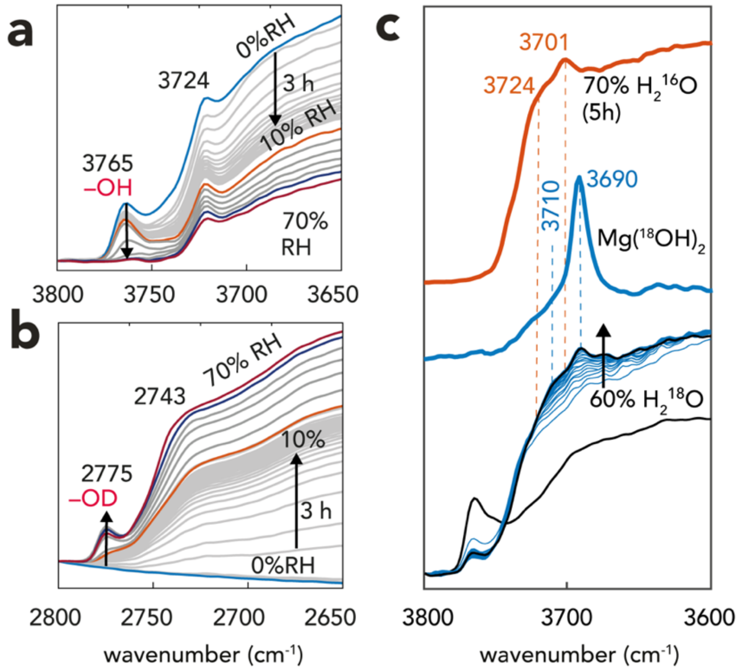

Fig S8. Isotopic exchange reveals OH groups on periclase and newly-grown nanosheets. Response of the (a) O-H stretching region to exposure of D_2O vapor (1-h equilibrated for each 10%-RH-increment from 10% to 70% RH), and (b) corresponding isotopic shift resulting from deuteration of OH groups. The spectra showed were selected after 1-h equilibrium of each step. Here, surface OH groups are readily deuterated while bulk groups are not in the reacted timeframe (1-h for each RH step) and humidity of the flow. (c) Brucite growth in 60% RH of $H_2{}^{18}O$ for 4 h revealing growth of $Mg(^{18}OH)_2$ with the 9 cm^{-1} isotopically-shifted O-H stretching band to 3690 cm^{-1} (*cf.* reference spectrum in blue) This experiment proved that the isotopically-shifted 3721 cm^{-1} band to 3710 cm^{-1} (*cf.* spectrum of periclase reacted with 70% RH of $H_2{}^{16}O$ for 5h, in orange) is a brucite-like material. All spectra were normalized for absorbance of the dry unreacted MgO at 3600 cm^{-1}.

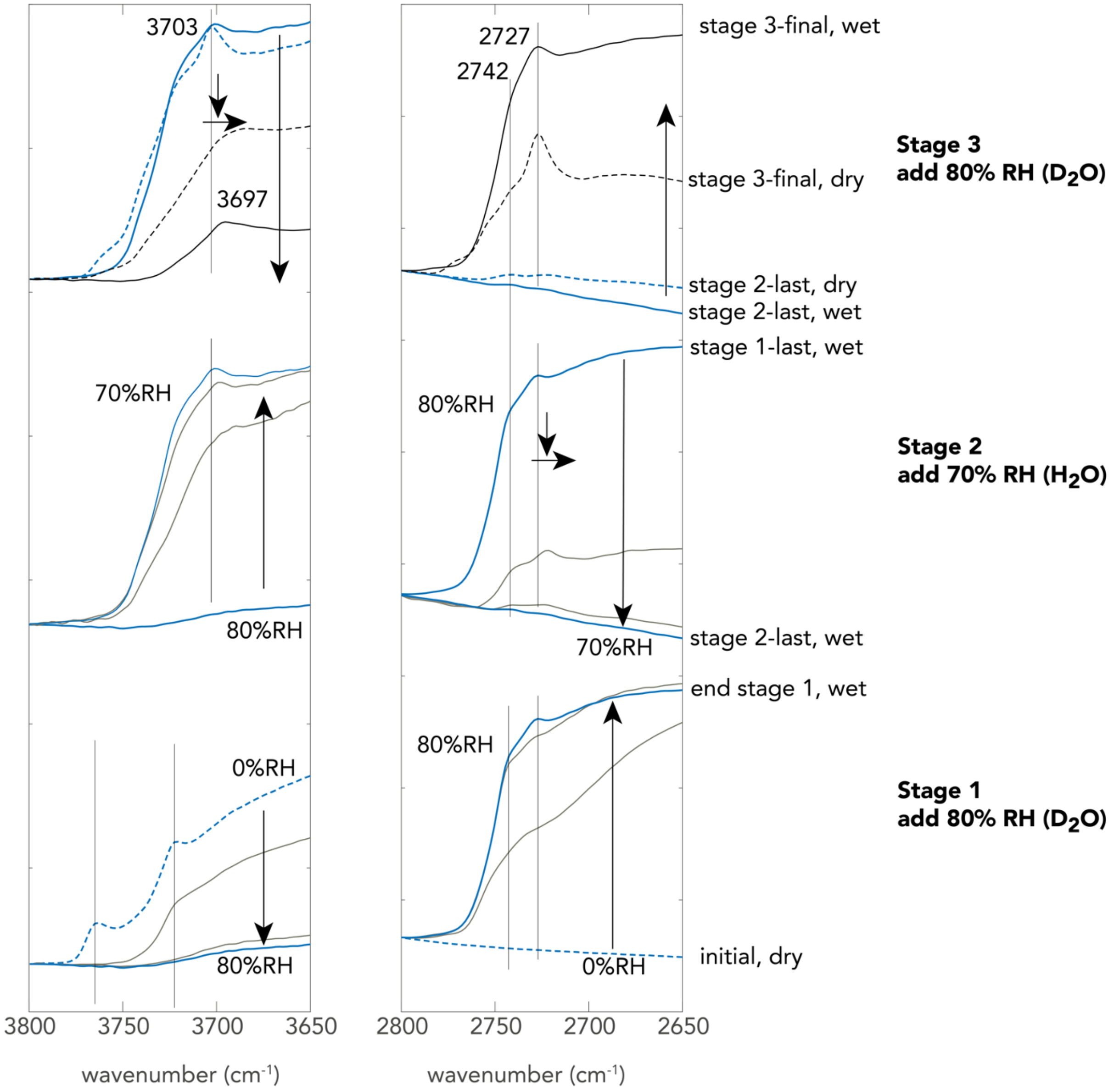

Fig S9. Isotopic exchange reveals surface OH groups on periclase. Pe5 was exposed to 80%RH (D_2O) in 2.6 h (stage 1), then switched to 70% RH (H_2O) for 1.3 h (stage 2), and finally deuterated at 80% RH for 1.0 h (stage 3). All spectra were normalized for absorbance of the dry unreacted MgO at 3600 cm^{-1}.

Table S1. Salient physicochemical properties periclase

Surface composition[a]	$Mg_{1.00}O_{0.84}(OH)_{0.46}(CO_3)_{0.05}$
Particle size[b] width (nm)	25-140
Average (nm)	77 ± 25
Crystallite size (nm)[c]	8.2 ± 0.4
Bulk composition	periclase
Specific surface area[d,e] (m^2/g)	154
t-plot micropore area[d](m^2/g)	1.8-1.9

a. For the top ~10 nm region probed by XPS.
b. From TEM
c. Rietveld refinement of XRD profiles.
d. From N_2(g) adsorption/desorption isotherm
e. Brunauer-Emmet-Teller (BET) analysis.
